# Cytotoxic Lesions of the Corpus Callosum: A Single-Center Case Series of Eight Patients With an Emphasis on Infectious Etiologies

**DOI:** 10.7759/cureus.110662

**Published:** 2026-06-11

**Authors:** Takeshi Yamashita, Kai Saito, Fukuko Matsumoto, Katsuyuki Yoshida, Michiko Adachi Matsuzawa, Hidenori Sanayama, Tamami Watanabe, Takahiko Fukuchi

**Affiliations:** 1 Division of General Medicine, Department of Comprehensive Medicine 1, Jichi Medical University Saitama Medical Center, Saitama, JPN

**Keywords:** anaerobic bacteria, cloccs, cytotoxic lesions of the corpus callosum, fusobacterium nucleatum, neuroimaging, reversible brain lesions

## Abstract

Background and aim

Cytotoxic lesions of the corpus callosum (CLOCCs) are transient lesions characterized by restricted diffusion on MRI and are associated with various infectious and noninfectious conditions. However, the clinical spectrum of infection-related CLOCCs, particularly those associated with bacterial infections, remains incompletely defined. This study aimed to describe the clinical, etiological, and neuroimaging characteristics of CLOCCs diagnosed at a single center, encompassing both infectious and noninfectious etiologies, with particular attention to infection-related cases.

Methods

We retrospectively reviewed patients diagnosed with CLOCCs at Jichi Medical University Saitama Medical Center between January 2016 and May 2025. Patients were identified by searching institutional radiology reports and electronic medical records for callosal lesions, followed by review of the corresponding records. Clinical characteristics, underlying etiologies, laboratory findings, MRI features, treatment, follow-up imaging, and clinical outcomes were extracted from medical records.

Results

Eight patients were identified (median age, 47.5 years; range, 12-70; five male and three female). Five patients had infection-related CLOCCs, including bacteremia, meningoencephalitis, and COVID-19-associated myocarditis. One patient had *Fusobacterium nucleatum* bacteremia, which, to the best of our knowledge, has not been well documented in association with CLOCCs. In all eight patients, the lesion was confined to the splenium of the corpus callosum. All patients showed hyperintensity on diffusion-weighted imaging, and six showed corresponding hypointensity on apparent diffusion coefficient maps. Follow-up MRI was available in seven patients (performed at a median of 17 days; range, 14-50 days); six showed complete resolution, whereas one showed near-complete resolution with faint residual fluid-attenuated inversion recovery hyperintensity. Seven patients improved clinically; one patient died during hospitalization from infection-related complications unrelated to the corpus callosum lesion, which had already resolved on follow-up MRI.

Conclusions

In this single-center case series, infection-related etiologies accounted for most of the cases. The association with *F. nucleatum* bacteremia, based on a single case, represents a possible rather than established addition to the bacterial infections reported in CLOCCs and should be regarded as hypothesis-generating. Early recognition of the characteristic MRI findings, evaluation for underlying infectious causes, and treatment directed at the underlying condition may support favorable clinical and radiological outcomes.

## Introduction

Cytotoxic lesions of the corpus callosum (CLOCCs) are transient lesions of the corpus callosum that typically show restricted diffusion on MRI and are associated with a wide range of conditions, including infections, metabolic disorders, malignancies, and drug-induced toxicities [1]. The underlying pathophysiology is thought to involve cytotoxic edema formation through complex cellular and cytokine-mediated mechanisms, such as the release of inflammatory cytokines (e.g., IL-1 and IL-6) from macrophages, recruitment of T cells, disruption of the blood-brain barrier, production of tumor necrosis factor-alpha (TNF-α), and astrocyte activation [1]. The predilection for the corpus callosum, particularly the splenium, is believed to result from its high density of glutamate receptors, supporting an excitotoxicity hypothesis in cytokine-mediated neuronal injury [2].

Among infectious causes, bacterial meningitis has been frequently reported in association with CLOCCs. CLOCCs have been observed in cases of *Streptococcus pneumoniae *and *Staphylococcus aureus *meningitis [2,3]. Despite the recognized association between bacterial infection and CLOCCs, cases associated with anaerobic bacteria, particularly *Fusobacterium *species, appear to be rare and remain poorly documented.

*Fusobacterium nucleatum *is a Gram-negative anaerobic bacillus that colonizes the human oral cavity and GI tract. Although often regarded as a commensal organism, it is increasingly recognized as an opportunistic pathogen capable of causing invasive systemic infections [4]. CNS infections caused by *F. nucleatum*, including brain abscesses and ventriculitis, have been reported, although they are rare [5-9]. However, CLOCCs associated with *F. nucleatum* bacteremia have not been well documented.

Recent systematic reviews have provided a clearer understanding of CLOCC epidemiology. A large meta-analysis including 1,353 cases from 324 studies reported a broad etiological spectrum, encompassing toxic, infectious (viral and bacterial), metabolic, vascular, neoplastic, and traumatic causes [2]. Although CLOCCs share these common pathophysiological features [1,2], it is useful to distinguish infection-associated cases from those arising in inflammatory or other systemic noninfectious conditions, because the underlying trigger guides clinical evaluation and management. In the present series, this distinction is reflected by the inclusion of infection-related cases alongside noninfectious etiologies, including Kawasaki disease, which we regarded as an inflammatory, noninfectious condition, as well as aortic dissection and cerebral infarction. Since the COVID-19 pandemic, multiple reports have described CLOCCs in patients with SARS-CoV-2 infection [10,11].

The terminology for transient splenial and callosal lesions has evolved over time. Terms such as “mild encephalitis/encephalopathy with reversible splenial lesion” (MERS) and “reversible splenial lesion syndrome” (RESLES) have increasingly been subsumed under the broader concept of CLOCCs, reflecting the recognition that lesions may extend beyond the splenium, may be associated with moderate or severe encephalopathy, and are not always fully reversible [12].

The primary aim of this study was to provide a descriptive characterization of the clinical features, etiologies, neuroimaging findings, and outcomes of patients with CLOCCs diagnosed at our institution over a nine-year period. As a secondary aim, we focused on the infection-related subset of this cohort and described a case of CLOCCs associated with *F. nucleatum* bacteremia, an association that, to the best of our knowledge, has not been previously reported. Through this descriptive case series, we sought to draw attention to bacterial, including anaerobic, infections as possible triggers of CLOCCs, while recognizing that a small retrospective series can indicate associations rather than establish causal relationships.

## Materials and methods

Study design

This was a retrospective, single-center observational study of patients diagnosed with CLOCCs at Jichi Medical University Saitama Medical Center between January 2016 and May 2025. The study aimed to characterize the clinical features, etiologies, neuroimaging findings, treatments, and outcomes of CLOCCs, with particular emphasis on infection-related cases.

Inclusion and exclusion criteria

Patients were identified by searching both institutional radiology reports and electronic medical records from the study period using a Japanese-language keyword equivalent to “splenial lesion of the corpus callosum.” The corresponding records and images were then reviewed to determine eligibility according to the inclusion and exclusion criteria described below. Patients of all ages were included if they had MRI findings compatible with CLOCCs, including a lesion involving the corpus callosum, hyperintensity on diffusion-weighted imaging (DWI), and available apparent diffusion coefficient (ADC) maps, together with sufficient clinical information, defined as documentation of the clinical presentation and symptoms, the presumed etiology or underlying disease, the treatment administered, and the clinical outcome. Restricted diffusion on ADC maps was assessed descriptively but was not required for inclusion, because CLOCCs may show variable ADC findings depending on lesion timing and severity. Reversibility on follow-up MRI was considered supportive of the diagnosis but was not required for inclusion when follow-up imaging was unavailable. Patients were excluded if MRI findings were incomplete or if the corpus callosum lesion was better explained by another established diagnosis, such as tumor, demyelinating disease, traumatic injury, chronic infarction, or a preexisting structural corpus callosum lesion.

Data collection

Medical records were retrospectively reviewed for demographic information, clinical presentation, underlying diseases, laboratory findings, microbiological results, presumed etiology, treatment modalities, hospital course, neurological outcome, and survival status. Clinical improvement was defined as survival to discharge or rehabilitation transfer without neurological sequelae attributable to CLOCCs. MRI findings were systematically evaluated for lesion location, signal characteristics on DWI, ADC, and fluid-attenuated inversion recovery (FLAIR), and radiological resolution on follow-up MRI when available. Infection-related CLOCCs were defined as cases in which the corpus callosum lesion was detected during an active, clinically diagnosed and treated infection, supported by microbiological evidence (blood culture, CSF culture, or nucleic acid amplification testing) or by clinical findings. Of the five infection-related cases, four (Cases 1, 4, 7, and 8) met microbiological criteria, whereas Case 2 was classified clinically as meningoencephalitis; no causative organism was identified, but CSF abnormalities, the clinical course of meningoencephalitis under antimicrobial and antiviral therapy, and subsequent radiological resolution of the corpus callosum lesion supported this classification.

Imaging protocol

All patients underwent brain MRI using a 1.5-T or 3.0-T scanner. The initial MRI was performed at 3.0 T in four patients (Cases 1, 4, 5, and 6) and at 1.5 T in four patients (Cases 2, 3, 7, and 8); follow-up examinations were performed at the same field strength as the initial study except in Case 6 (initial 3.0 T, follow-up 1.5 T). Because the study was retrospective and some examinations were performed at referring institutions, the field strength and scanners were not standardized. Standard sequences included T1-weighted imaging, T2-weighted imaging, FLAIR imaging, DWI with a b-value of 1000 s/mm², and ADC mapping; additional sequences, including MR angiography and T2*-weighted imaging, were performed according to clinical indications. Brain MRI findings were interpreted as part of routine clinical care; in six cases, the diagnosis was based on formal radiology reports, and in two cases (Cases 1 and 7), the imaging was evaluated by neurologists. No blinded, study-specific independent re-review or formal consensus reading was performed.

Statistical analysis

Descriptive statistics were used to summarize patient characteristics. Continuous variables are presented as medians and ranges, and categorical variables are presented as numbers. Because of the small sample size and descriptive nature of the study, formal statistical comparisons were not performed.

Ethical considerations

This study was conducted in accordance with the Declaration of Helsinki and was approved by the Institutional Review Board (IRB) of Jichi Medical University Saitama Medical Center (approval number: S25-043; approval date: August 12, 2025). The requirement for individual informed consent was waived by the IRB because of the retrospective design and the use of anonymized clinical data and images.

## Results

Patient demographics

Eight patients with CLOCCs were identified during the study period (median age, 47.5 years; range, 12-70; five male and three female). The most common clinical features were fever, which occurred during the hospital course in all eight patients, and altered mental status, present in six; no patient had seizures, and CNS infection (meningoencephalitis) was identified in one. Five patients were classified as having infection-related CLOCCs, comprising bacteremia, meningoencephalitis, and COVID-19-associated myocarditis, and three as having noninfectious etiologies (aortic dissection, Kawasaki disease, and atherothrombotic cerebral infarction). In all eight patients, the lesion was confined to the splenium of the corpus callosum. Treatment was directed at the underlying condition in every case, most commonly antimicrobial therapy in the infection-related cases. A follow-up MRI was performed in seven patients at a median of 17 days (range, 14-50 days), showing complete resolution in six and near-complete resolution in one. Seven patients improved clinically; one patient with meningoencephalitis died during hospitalization from infection-related complications unrelated to the corpus callosum lesion (Table 1).

**Table 1:** Summary of eight cases of CLOCCs diagnosed at our institution This table summarizes demographic data, etiologies, lesion location, treatments, MRI findings, MRI timing, and outcomes. All MRI timing is expressed as a hospital day. ^*^ In Case 3, the initial MRI was performed on hospital day 36 during evaluation of postoperative complications. ADC, apparent diffusion coefficient; CLOCCs, cytotoxic lesions of the corpus callosum; DWI, diffusion-weighted imaging; FLAIR, fluid-attenuated inversion recovery

Case	Age	Sex	Disease	Etiology	Location	Treatment	MRI findings	Initial MRI	Follow-up MRI	Outcome
1	30	M	*Streptococcus* species bacteremia	Infection-related	Splenium	IV antibiotics	DWI (high), ADC (low), FLAIR (no significant abnormality)	Day 2	Day 44	Improved after six weeks of antibiotic therapy
2	70	F	Meningoencephalitis	Infection-related	Splenium	Broad-spectrum antibiotics and acyclovir	DWI (high), ADC (low), FLAIR (no significant abnormality)	Day 3	Day 17	Died during hospitalization on day 43
3	47	M	Stanford type A aortic dissection	Noninfectious	Splenium	Total arch replacement	DWI (high), ADC (no marked reduction), FLAIR (high)	Day 36^*^	Day 50	Improved; transferred for rehabilitation on day 56
4	19	M	COVID-19-associated fulminant myocarditis	Infection-related	Splenium	Dexamethasone and heparin	DWI (high), ADC (low), FLAIR (high)	Day 0 (pre-admission)	Day 14	Improved; discharged on day 18
5	12	M	Kawasaki disease	Noninfectious	Splenium	Immunoglobulin, prednisolone, and infliximab	DWI (high), ADC (no marked reduction), FLAIR (high)	Day 3	Day 15	Improved; discharged on day 18
6	50	F	Left putamen and corona radiata atherothrombotic cerebral infarction	Noninfectious	Splenium	Tissue plasminogen activator and dual antiplatelet therapy	DWI (high), ADC (low), FLAIR (high)	Day 7	Day 21	Improved; transferred for rehabilitation on day 23
7	50	F	*Streptococcus dysgalactiae* subsp. *equisimilis* bacteremia	Infection-related	Splenium	Antibiotic therapy	DWI (high), ADC (low), FLAIR (high)	Day 1	Not performed	Improved; discharged after two weeks
8	48	M	*Fusobacterium nucleatum* bacteremia	Infection-related	Splenium	Antibiotics changed from ceftriaxone to ampicillin-sulbactam	DWI (high), ADC (low), FLAIR (no significant abnormality)	Day 2	Day 14	Improved; discharged without neurological sequelae

Individual case descriptions

Case 1: 30-Year-Old Man With Streptococcus Species Bacteremia

**Figure 1:**
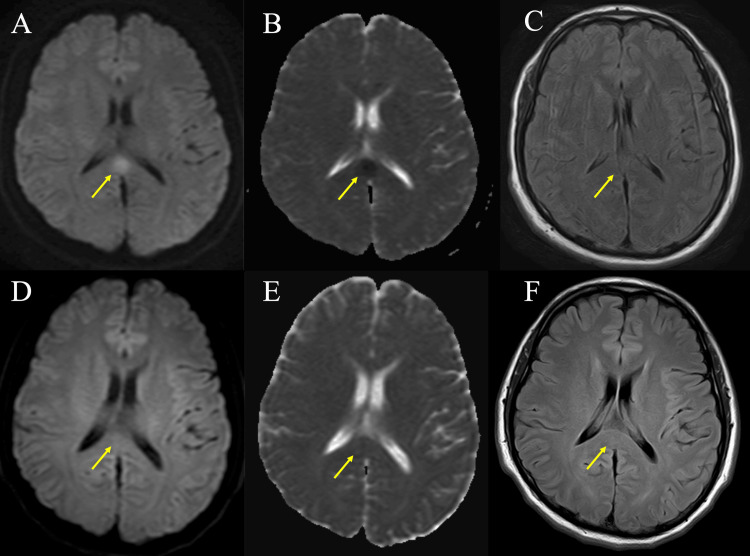
Case 1: MRI findings of CLOCCs (A) DWI shows a high-signal-intensity lesion in the splenium of the corpus callosum (yellow arrow). (B) The ADC map shows a corresponding low-signal-intensity lesion, indicating restricted diffusion (yellow arrow). (C) FLAIR imaging shows no significant signal abnormality in the corpus callosum (yellow arrow). (D-F) Follow-up MRI shows complete resolution of the corpus callosum lesion on DWI (D), ADC (E), and FLAIR (F). ADC, apparent diffusion coefficient; CLOCCs, cytotoxic lesions of the corpus callosum; DWI, diffusion-weighted imaging; FLAIR, fluid-attenuated inversion recovery

A 30-year-old man with a five-year history of type 2 diabetes presented after collapsing while cycling home from dental treatment. He had experienced a toothache for three days and developed chills and tremors immediately after the dental procedure. On arrival, he was unconscious. Brain MRI revealed high signal intensity on DWI and low signal intensity on ADC in the corpus callosum (Figure 1). Blood cultures grew *Streptococcus *species (species-level identification was not performed), and echocardiography showed vegetations consistent with infective endocarditis, with dental caries considered the likely source. The patient recovered after six weeks of IV antibiotic therapy.

Case 2: 70-Year-Old Woman With Meningoencephalitis

**Figure 2:**
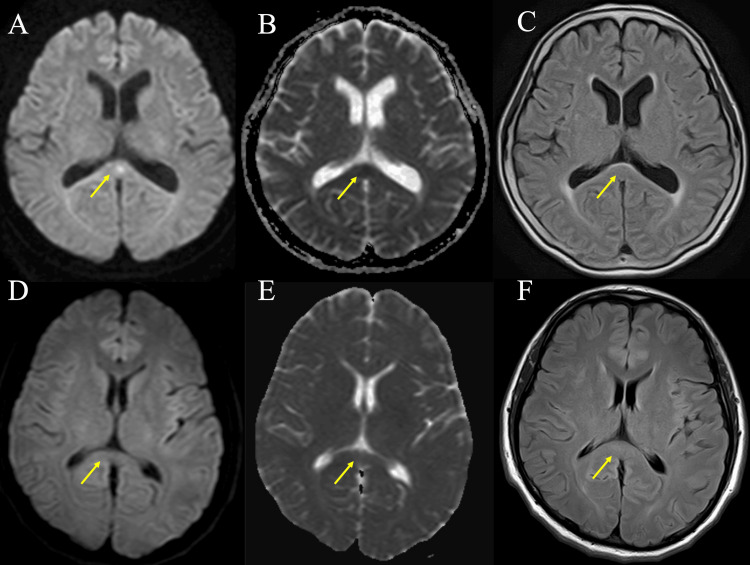
Case 2: MRI findings of CLOCCs (A) DWI shows a high-signal-intensity lesion in the splenium of the corpus callosum (yellow arrow). (B) The ADC map shows a corresponding low-signal-intensity lesion, consistent with cytotoxic edema (yellow arrow). (C) FLAIR imaging shows no significant signal abnormality in the corpus callosum (yellow arrow). (D-F) Follow-up MRI shows complete resolution of the corpus callosum lesion on DWI (D), ADC (E), and FLAIR (F). ADC, apparent diffusion coefficient; CLOCCs, cytotoxic lesions of the corpus callosum; DWI, diffusion-weighted imaging; FLAIR, fluid-attenuated inversion recovery

A 70-year-old woman presented with persistent high fever (39°C), headache, dizziness, and vomiting despite outpatient antibiotic treatment. Physical examination revealed nuchal rigidity and altered mental status. CSF analysis showed an opening pressure of 16 cm CSF, a protein level of 312 mg/dL, a glucose level of 38 mg/dL, and pleocytosis (380 cells/µL; 152 polymorphonuclear and 228 mononuclear cells), with 67 red blood cells/µL. CSF cultures and nucleic acid amplification testing did not identify a causative organism. Brain MRI on day 3 demonstrated high signal intensity on DWI and low signal intensity on ADC in the corpus callosum (Figure 2). She was treated empirically with broad-spectrum antibiotics and acyclovir, and a follow-up MRI on day 17 showed complete resolution of the corpus callosum lesion. She subsequently developed catheter-related *Pseudomonas *bacteremia and *Candida *fungemia, unrelated to the corpus callosum lesion. She died from ventricular fibrillation on day 43; autopsy revealed infective endocarditis and pulmonary embolism, indicating an infection-related rather than neurological cause of death.

Case 3: 47-Year-Old Man With Stanford Type A Aortic Dissection

**Figure 3:**
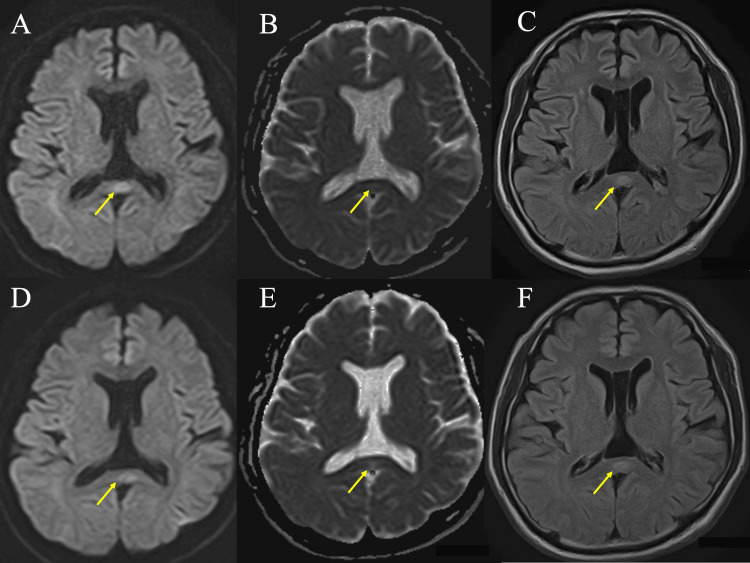
Case 3: MRI findings of CLOCCs (A) DWI shows a high-signal-intensity lesion in the corpus callosum (yellow arrow). (B) The ADC map shows no marked signal reduction in the corresponding region (yellow arrow). (C) FLAIR imaging shows high signal intensity in the affected region (yellow arrow). (D-F) Follow-up MRI shows marked improvement of the corpus callosum lesion, with no marked residual abnormality on DWI (D), slightly increased signal intensity on the ADC map (E), and faint residual hyperintensity on FLAIR imaging (F). ADC, apparent diffusion coefficient; CLOCCs, cytotoxic lesions of the corpus callosum; DWI, diffusion-weighted imaging; FLAIR, fluid-attenuated inversion recovery

A 47-year-old man presented with acute chest and back pain. Contrast-enhanced CT revealed Stanford type A aortic dissection extending from the ascending aorta to the left common iliac artery. He underwent emergent total arch replacement. Postoperatively, he required prolonged mechanical ventilation and dialysis and showed altered consciousness. He subsequently developed ventilator-associated pneumonia with fever during mechanical ventilation. On hospital day 36, brain MRI revealed high signal intensity on DWI in the corpus callosum. The corresponding ADC map, however, did not show marked signal reduction (Figure 3). His consciousness gradually improved, and a follow-up MRI on hospital day 50 showed marked improvement of the corpus callosum lesion, with faint residual hyperintensity on FLAIR imaging and slightly increased signal intensity on the ADC map. He was transferred for rehabilitation on hospital day 56.

Case 4: 19-Year-Old Man With COVID-19-Associated Fulminant Myocarditis

**Figure 4:**
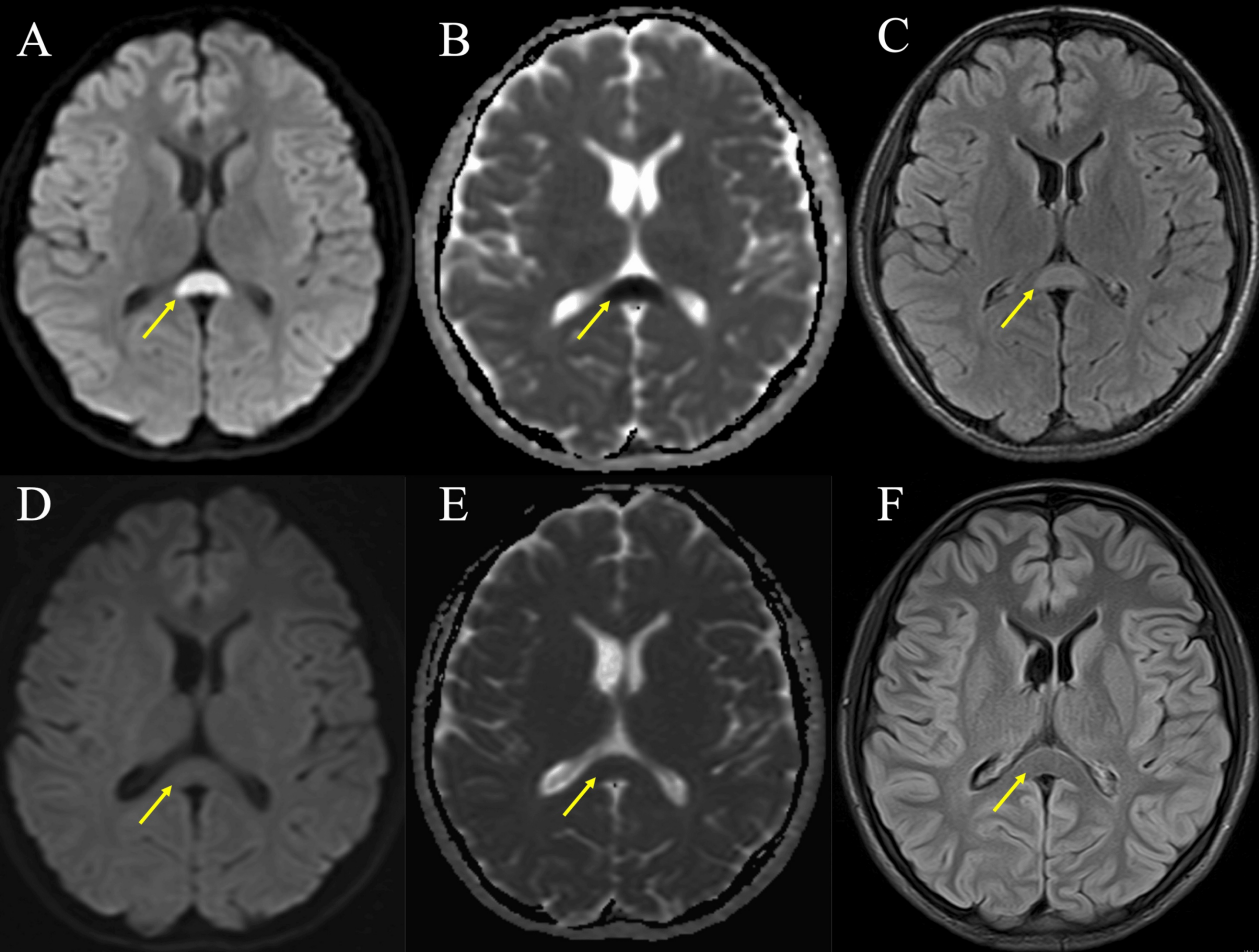
Case 4: MRI findings of CLOCCs (A) DWI shows a high-signal-intensity lesion in the splenium of the corpus callosum (yellow arrow). (B) The ADC map shows low signal intensity, consistent with cytotoxic edema (yellow arrow). (C) FLAIR imaging shows high signal intensity in the corpus callosum (yellow arrow). (D-F) Follow-up MRI shows complete resolution of the corpus callosum abnormalities. ADC, apparent diffusion coefficient; CLOCCs, cytotoxic lesions of the corpus callosum; DWI, diffusion-weighted imaging; FLAIR, fluid-attenuated inversion recovery

A 19-year-old man presented with high fever (40°C) and vomiting three days after symptom onset. He tested positive for SARS-CoV-2 and developed fulminant myocarditis with the ejection fraction declining from 40% to 25%. Brain MRI, obtained to evaluate thromboembolic complications of COVID-19, showed high signal intensity on DWI and low signal intensity on ADC in the corpus callosum (Figure 4). This lesion was considered most likely related to systemic inflammation associated with SARS-CoV-2 infection. Myocardial biopsy revealed lymphocytic infiltration. He was treated with dexamethasone and heparin. Follow-up MRI on day 14 showed complete resolution, and he was discharged on day 18.

Case 5: 12-Year-Old Boy With Kawasaki Disease

**Figure 5:**
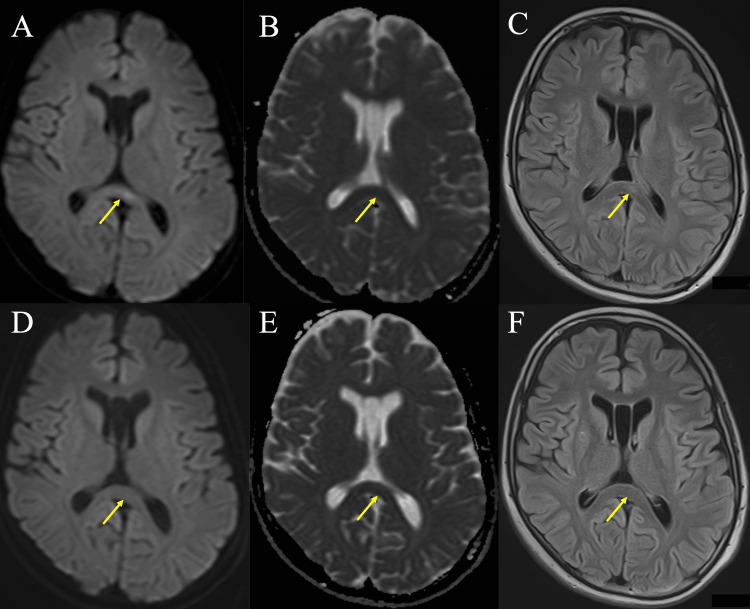
Case 5: MRI findings of CLOCCs (A) DWI shows a high-signal-intensity lesion in the splenium of the corpus callosum (yellow arrow). (B) The ADC map shows no marked signal reduction in the corresponding region (yellow arrow). (C) FLAIR imaging shows high signal intensity in the corpus callosum (yellow arrow). (D-F) Follow-up MRI shows complete resolution of the corpus callosum abnormalities. ADC, apparent diffusion coefficient; CLOCCs, cytotoxic lesions of the corpus callosum; DWI, diffusion-weighted imaging; FLAIR, fluid-attenuated inversion recovery

A 12-year-old boy presented with six days of fever (38.3°C), headache, and progressive erythematous rash. On admission, he exhibited conjunctival injection, strawberry tongue, cervical lymphadenopathy, and altered mental status with hallucinations and disorientation. Laboratory tests showed marked inflammation (white blood cell count, 14,500/µL; C-reactive protein, 19.5 mg/dL). Brain MRI on day 3 revealed high signal intensity on DWI in the corpus callosum, whereas the ADC map showed no marked signal reduction (Figure 5). He was treated with IVIG, infliximab, and corticosteroids. Follow-up MRI on day 15 showed complete resolution, and he was discharged on day 18.

Case 6: 50-Year-Old Woman With Atherothrombotic Cerebral Infarction

**Figure 6:**
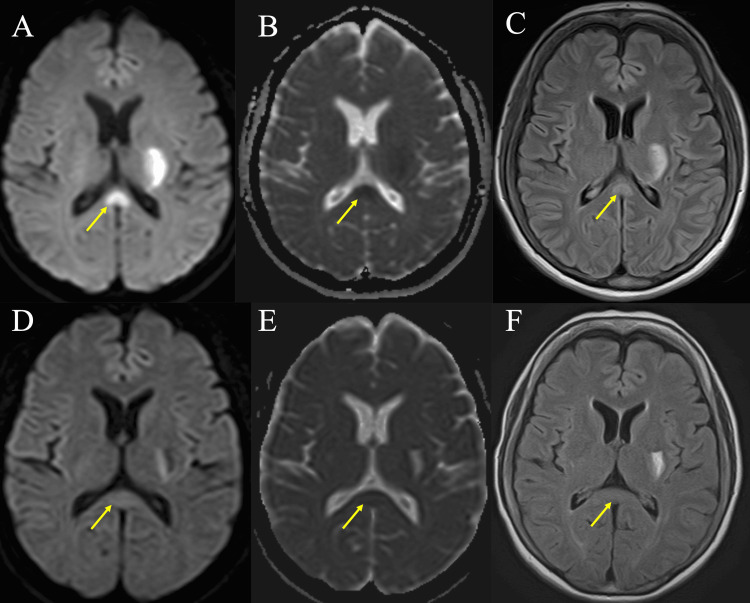
Case 6: MRI findings of CLOCCs (A) DWI on day 7 shows a high-signal-intensity lesion in the corpus callosum (yellow arrow). The initial MRI showed left putamen and corona radiata infarction, which is not shown. (B) The ADC map shows corresponding low signal intensity (yellow arrow). (C) FLAIR imaging shows high signal intensity in the corpus callosum (yellow arrow). (D-F) Follow-up MRI shows complete resolution of the corpus callosum lesion on all sequences. ADC, apparent diffusion coefficient; CLOCCs, cytotoxic lesions of the corpus callosum; DWI, diffusion-weighted imaging; FLAIR, fluid-attenuated inversion recovery

A 50-year-old woman with Graves’ disease presented with transient right-sided weakness and dysarthria. Brain MRI showed an acute infarction in the left putamen and corona radiata with high signal intensity on DWI and low signal intensity on ADC. She received tissue plasminogen activator followed by dual antiplatelet therapy. A low-grade fever developed during hospitalization. The initial MRI showed no corpus callosum lesion; follow-up MRI on day 7 additionally revealed high signal intensity on DWI, low signal intensity on ADC, and high signal intensity on FLAIR in the corpus callosum, consistent with CLOCCs (Figure 6). The corpus callosum lesion resolved on repeat imaging on day 21. She was transferred for rehabilitation on day 23.

Case 7: 50-Year-Old Woman With Streptococcus dysgalactiae subsp. equisimilis Bacteremia

**Figure 7:**
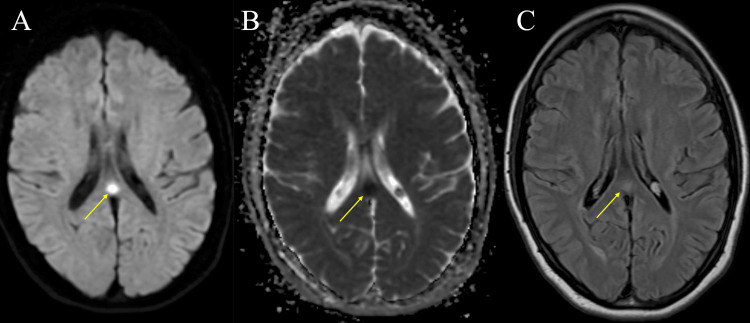
Case 7: MRI findings of CLOCCs (A) DWI shows a high-signal-intensity lesion in the corpus callosum (yellow arrow). (B) The ADC map shows a corresponding low-signal-intensity lesion, indicating restricted diffusion (yellow arrow). (C) FLAIR imaging shows high signal intensity in the corpus callosum (yellow arrow). A follow-up MRI was not available for this case. ADC, apparent diffusion coefficient; CLOCCs, cytotoxic lesions of the corpus callosum; DWI, diffusion-weighted imaging; FLAIR, fluid-attenuated inversion recovery

A 50-year-old woman with chronic myeloid leukemia on dasatinib and a previous colectomy for ulcerative colitis developed fever, arthralgia, and anorexia four days before admission. On arrival, she had an altered mental status. Blood cultures grew *Streptococcus dysgalactiae *subsp. *equisimilis*. Brain MRI demonstrated high signal intensity on DWI, low signal intensity on ADC, and high signal intensity on FLAIR in the corpus callosum (Figure 7). CSF showed mild mononuclear pleocytosis. She responded well to antibiotic therapy and was discharged after two weeks. Because a follow-up MRI was not performed, radiological reversibility could not be confirmed, and this case is therefore regarded as a probable rather than radiologically confirmed reversible CLOCC.

Case 8: 48-Year-Old Man With F. nucleatum Bacteremia

**Figure 8:**
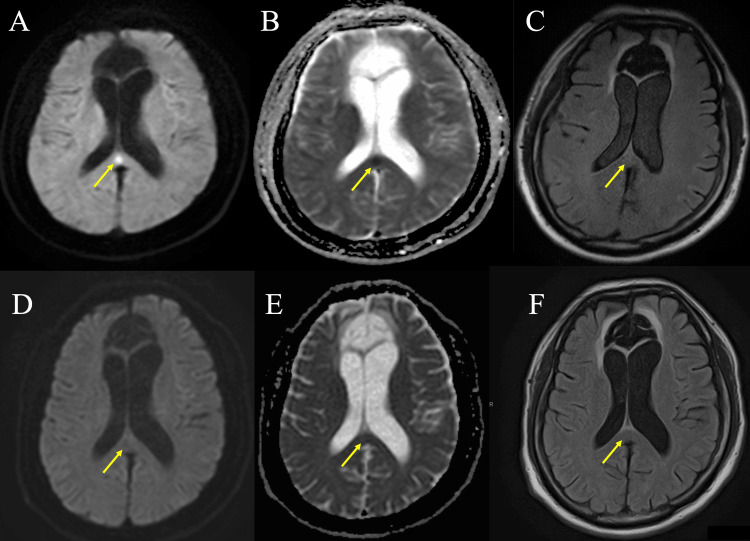
Case 8: MRI findings of CLOCCs (A) DWI shows a high-signal-intensity lesion in the corpus callosum (yellow arrow). Previous bilateral frontal lobe infarcts are also visible. (B) The ADC map shows a corresponding low-signal-intensity lesion, consistent with cytotoxic edema (yellow arrow). (C) FLAIR imaging shows no significant signal abnormality in the corpus callosum (yellow arrow). (D-F) Follow-up MRI shows complete resolution of the corpus callosum lesion on all sequences. ADC, apparent diffusion coefficient; CLOCCs, cytotoxic lesions of the corpus callosum; DWI, diffusion-weighted imaging; FLAIR, fluid-attenuated inversion recovery

A 48-year-old man with severe obesity (BMI, 49 kg/m²) and prior bilateral frontal infarctions presented with fever (40°C), altered consciousness, and right lower leg cellulitis. He had no known immunosuppressive disease, was not receiving immunosuppressive medications, and had no marked electrolyte abnormalities, including hyponatremia. Brain MRI revealed high signal intensity on DWI and low signal intensity on ADC in the corpus callosum without significant FLAIR signal abnormality (Figure 8). Blood cultures obtained on admission became positive for *F. nucleatum *on hospital day 10, prompting a change in antibiotic therapy from ceftriaxone to ampicillin-sulbactam. Although the patient had right lower leg cellulitis, the source of the bacteremia remained unclear; dental, GI, and CT evaluations for oral, GI, or malignant sources were unremarkable, and no definite portal of entry was identified. Follow-up MRI on hospital day 14 showed complete resolution, and he was discharged without neurological sequelae.

Imaging findings summary

All eight patients demonstrated high signal intensity on DWI in the corpus callosum. ADC findings varied: six patients showed low signal intensity, whereas two showed no marked signal reduction. FLAIR hyperintensity was observed in five patients. On follow-up imaging, the single patient without complete resolution showed faint residual FLAIR hyperintensity and slightly increased ADC signal intensity.

## Discussion

In this single-center case series of eight patients with CLOCCs, infection-related etiologies accounted for five cases, with the remaining three attributable to noninfectious conditions (aortic dissection, Kawasaki disease, and atherothrombotic infarction). One patient had* F. nucleatum* bacteremia, which, to the best of our knowledge based on a literature search, has not been previously reported in association with CLOCCs. However, as this observation is based on a single case, it should be regarded as a possible rather than an established association. The predominance of infection-related cases in this series reflects the case composition of a single tertiary care center and should not be interpreted as indicating the frequency of infectious etiologies among CLOCCs in general.

The case associated with *F. nucleatum* bacteremia may help expand the recognized spectrum of bacterial infections associated with CLOCCs. Previous reports have described CLOCCs in association with bacterial meningitis and other bacterial infections, including cases caused by *S. pneumoniae *and *S. aureus *[2,3]. In our series, the bacterial cases were associated with bacteremia rather than direct CNS infection. Our findings suggest that anaerobic organisms may also be considered as possible infectious triggers, although causality cannot be established from a single case.

*F. nucleatum *is a Gram-negative anaerobic bacillus that colonizes the oral cavity and GI tract and is increasingly recognized as an opportunistic pathogen capable of causing invasive systemic infections [4]. Rare CNS infections caused by *F. nucleatum*, including brain abscesses and ventriculitis, have been reported [5-9]. In the present case, *F. nucleatum* bacteremia occurred in close temporal association with CLOCCs, and the lesion resolved after antimicrobial therapy. This temporal relationship suggests a possible association but does not prove direct causality. The delayed culture positivity on day 10 reflected the slow growth of this anaerobic organism rather than late-onset infection, as the patient was treated as having an infection from admission, and this does not alter the temporal relationship with CLOCCs.

The pathogenesis of CLOCCs in infectious settings remains incompletely understood. Proposed mechanisms include systemic inflammation, cytokine-mediated toxicity, blood-brain barrier dysfunction, and microvascular injury leading to cytotoxic edema [1,2]. The corpus callosum, particularly the splenium, may be especially vulnerable because of its high concentration of glutamate receptors, which may predispose it to excitotoxic injury during systemic inflammation. Among these mechanisms, cytokine-mediated toxicity and systemic inflammation appear most relevant to the present series, in which infection-related cases predominated. In bacteremia and other systemic infections, circulating proinflammatory cytokines such as IL-1, IL-6, and TNF-α, together with blood-brain barrier dysfunction, are thought to promote cytotoxic edema in the splenium [1,2]. This framework offers a plausible link between bacteremia, including the anaerobic infection observed in our cohort, and the development of CLOCCs, although the precise pathway cannot be confirmed from a descriptive case series.

Neuroimaging played a critical role in the diagnosis of CLOCCs. All patients demonstrated DWI hyperintensity, and six patients showed corresponding ADC hypointensity, findings consistent with cytotoxic edema. The two patients without marked ADC signal reduction may reflect differences in imaging timing, lesion severity, or heterogeneity in the underlying pathophysiology. In these cases, the diagnosis was supported by DWI hyperintensity, the clinical context, lesion reversibility on follow-up imaging, and exclusion of alternative causes, consistent with the recognition that restricted diffusion, although characteristic, is not invariably present in CLOCCs. In one patient, follow-up MRI showed slightly increased ADC signal intensity despite marked improvement on DWI and FLAIR, which may reflect the recovery phase of cytotoxic edema or a mixed cytotoxic and vasogenic component. Follow-up MRI, when available, helped confirm the transient nature of the corpus callosum lesions.

Differential diagnoses for corpus callosum lesions include Marchiafava-Bignami disease, demyelinating disorders, lymphoma, acute disseminated encephalomyelitis, ischemic infarction, and traumatic injury. The combination of characteristic diffusion restriction, clinical context, and radiological resolution on follow-up imaging helps distinguish CLOCCs from these other entities. In the present cases, these alternatives were considered unlikely on the basis of splenial location, lesion reversibility where available, and the clinical course, and lesions that proved to represent other entities, such as lymphoma, were excluded during case identification.

Treatment was directed at the underlying condition rather than the corpus callosum lesion itself, and most patients improved clinically, supporting the generally favorable course of CLOCCs; the single death was attributable to infection-related complications rather than to the callosal lesion, which had resolved on follow-up imaging. When CLOCCs are identified in an appropriate clinical context, bacterial infection, including anaerobic infection, should be considered; early MRI recognition and microbiological evaluation may facilitate treatment directed at the underlying cause, and follow-up MRI can help document lesion resolution.

This study has several limitations. First, this was a retrospective single-center case series with a small sample size, limiting generalizability. Second, the etiologies were heterogeneous, making common pathophysiological mechanisms difficult to establish; etiologic attribution was also uncertain in some patients with complex systemic illness, in whom infection, inflammation, vascular injury, or critical illness may all have contributed. Third, the association between *F. nucleatum* bacteremia and CLOCCs was based on temporal and clinical correlation, and direct causality cannot be inferred. Fourth, MRI protocols and timing were not standardized; because CLOCCs evolve over time, this may have contributed to variability in ADC and FLAIR findings and affected the assessment of lesion resolution. Fifth, one patient (Case 7) did not undergo follow-up MRI, so reversibility could not be confirmed, and this case is considered a probable rather than radiologically confirmed CLOCC; long-term neurological outcomes were also not uniformly available. Sixth, as a tertiary care center, selection bias toward more severe or complex cases may have been present. Finally, case identification used a single splenium-focused keyword (“splenial lesion of the corpus callosum”), which may have missed lesions in other callosal regions or those described using different terminology, introducing ascertainment bias; image interpretation also relied on routine clinical reads rather than blinded or consensus review, which may have introduced variability in lesion classification.

## Conclusions

This single-center case series describes eight patients with CLOCCs, most of whom had infection-related etiologies. One case was associated with* F. nucleatum *bacteremia; based on a single case with temporal association only, anaerobic bacteremia should be regarded as a possible rather than an established trigger. Recognition of characteristic MRI findings, evaluation for underlying infectious causes, and treatment directed at the underlying condition may support favorable clinical and radiological outcomes. As a small retrospective series, this study can suggest associations but cannot establish causality, and the findings are hypothesis-generating. Further studies are needed to clarify the mechanisms linking systemic infection, inflammatory responses, and cytotoxic callosal lesions.
